# Chronic Disease Self-Management Challenges among Rural Women Living with HIV/AIDS in Prakasam, Andhra Pradesh, India: A Qualitative Study

**DOI:** 10.1177/2325958218773768

**Published:** 2018-05-14

**Authors:** Benissa E. Salem, Yvita Bustos, Chidyaonga Shalita, Jordan Kwon, Padma Ramakrishnan, Kartik Yadav, Maria L. Ekstrand, Sanjeev Sinha, Adeline M. Nyamathi

**Affiliations:** 1 School of Nursing, University of California, Los Angeles, Los Angeles, CA, USA; 2 Psychology Department, Loyola University, Chicago, IL, USA; 3 Biology Department, Macalester College, Forest Lake, MN, USA; 4 School of Nursing, Azusa Pacific University, Montebello, CA, USA; 5 All India Institute of Medical Sciences (AIIMS), Nellore, India; 6 School of Nursing, University of California, Irvine, Irvine, CA, USA; 7 University of California, San Francisco School of Medicine, San Francisco, CA, USA; 8 All India Institute of Medical Sciences (AIIMS), Ansari Nagar, New Delhi, India; 9 School of Nursing, University of California, Irvine, Irvine, CA, USA

**Keywords:** HIV/AIDS, antiretroviral therapy, self-management, rural women, India

## Abstract

Rural women living with HIV/AIDS (WLHA) in India experience challenges self-managing HIV/AIDS in their rural communities. The purpose of this qualitative study was to explore factors influencing their care and antiretroviral treatment (ART) adherence. Themes that emerged from the qualitative focus groups among WLHA (N = 24) in rural Prakasam, Andhra Pradesh, India, included: (1) coming to know about HIV and other health conditions, (2) experiences being on ART, (3) challenges maintaining a nutritious diet, (4) factors affecting health care access and quality, and (5) seeking support for a better future. Chronic disease self-management in rural locales is challenging, given the number of barriers which rural women experience on a daily basis. These findings suggest a need for individual- and structural-level supports that will aid in assisting rural WLHA to self-manage HIV/AIDS as a chronic illness.

## Introduction

India has the third largest HIV-positive population globally, with 2.1 million people living with HIV (PLWH).^1,2^ In 2015, the national adult HIV prevalence among 15- to 49-year-olds was 0.26%,^3,4^ and among women, it was 0.22%.^3^ In South India, Andhra Pradesh is one of 4 high HIV prevalence states in the country.^5^ In 2015, Andhra Pradesh and Telangana had a total estimated HIV prevalence of 0.66% among 15- to 49-year-olds.^3,6^ However, among Andhra Pradesh antenatal clinic (ANC) attendees, the HIV prevalence was 0.41%.^7^ Within Andhra Pradesh, Prakasam is one of 23 districts^8,9^ and is in a coastal part of the state.^10^ While there is a paucity of HIV prevalence data from Prakasam district, between 2014 and 2015, in ANCs, the HIV prevalence ranged from 0.27% to 1.00%.^6^ General background of the literature and population is discussed in the subsequent paragraphs.

Women living with HIV/AIDS (WLHA) account for 39% of all cases in India.^2^ In India, heterosexual transmission is a major route of HIV transmission.^11,12^ A myriad of factors place women at increased risk of HIV transmission, which include spouses who migrate or spend long periods away from home,^13^ the lack of decision-making about sex, negotiating condom use,^14^ and intimate partner violence (IPV; eg, physical and sexual violence).^15^

Studies suggest that men who migrate or spend long periods away from home may engage in risky sexual behavior due to separation from their families.^16–18^ In a Mumbai-based study (N = 5130), data revealed that married men with wives in close proximity to area of origin and who were able to be mobile due to occupation had the highest sexual risk behaviors.^17^

A number of studies document challenges with negotiating condom use among women and their partners.^19^ Due to the need for economic stability, some women engage in transactional sex to provide support for their family.^19^ Beliefs about condom use are important to consider as data reveal that if a woman asks her partner to use a condom, he may lose respect for her due to mistrust in their relationship.^20^ Moreover, HIV/AIDS may not be openly discussed due to the taboo nature of the topic, therefore decreasing awareness of HIV and increasing vulnerability to infection.^21^ Finally, among women who experienced physical and sexual violence, findings revealed they had almost a 4 times higher HIV prevalence than participants who did not experience IPV (*P* = .01).^15^

### Challenges among WLHA Experience

For over a decade, National AIDS Control Organization has enabled first-line antiretroviral therapy (ART) to be free in India at all ART centers.^22^ As a corollary, the expansion of voluntary counseling and testing centers in Andhra Pradesh allowed for greater accessibility.^23^ Nevertheless, WLHA experience both public and private stigma.^24^ Stigma and discrimination impedes HIV disclosure to family and friends,^25,26^ quality and access to care,^27^ willingness to seek medical care,^28^ and early detection of HIV infection.^29^

In a qualitative study among mothers living with HIV/AIDS (MLH; N = 60, age range = 23-42), data revealed that disclosing HIV status was of great concern; in particular, MLH feared that their children would not be admitted into schools and they felt their children would also discriminate against them once they found out their status.^25^ Not only do women experience both stigma and discrimination, but also other challenges which rural WLHA experience that were physical, mental, financial, and transportation related.^30^ For WLHA living in Prakasam, additional challenges that can be experienced include environment factors; in particular, Prakasam is a district in the state of Andhra Pradesh, India, which has seasonal drought^31–33^ and it is plausible that challenges with accessing nutritious foods can be more difficult.

### Chronic Disease Self-Management Challenges

Chronic disease self-management (CDSM) can be generally understood as the ability to manage chronic health conditions, which may encompass cognitive symptom management (ie, depression, fatigue, sleep management, and so on).^34,35^ Relatedly, the ability to manage exercise, use medications, and access community resources is important in CDSM^34,35^; however, these challenges can abound for rural women and children living with HIV/AIDS.

When applied to rural WLHA, challenges to CDSM may include difficulty managing physical and mental health symptoms, accessing and adhering to ARV medication, food insecurity, and health care access. Treatment adherence, which can be defined as taking ARV medication as prescribed,^22^ is an ongoing challenge and an emergent theme which was generated among focus groups with rural WLHA. In particular, social and physical barriers such as HIV-related stigma have been noted.^36^ For PLWH, depression is a prevalent neuropsychiatric condition^37^ and a considerable issue as it is highly related to nonadherence to ART.^38^ Another critical area to explore is the relationship between food security, defined as when people have access to sufficient, safe, and nutritious food that meets their dietary needs,^39^ and ART adherence. Among PLWH, malnutrition has been associated with increased disease progression, higher mortality, and decreased response to ART.^40^ In resource-poor settings, food insecurity has been associated with increased opportunistic infections and hospitalizations.^41^

### Community-Based Self-Management Programs among Rural WLHA

Previous studies have addressed management of HIV/AIDS and have included the utilization of Activated Social Health Activists (ASHA), also known as lay health women, to deliver an intervention for rural women in Andhra Pradesh. Prior to delivering intervention trials, early studies focusing on understanding perceptions of rural WLHA and of having village women (ASHA) delivering the intervention were conducted,^30,42^ which led to future intervention trials.

The ASHA-Life (AL) intervention, a 6-month intervention, includes 6 program-specific sessions which included: 1) HIV/AIDS and managing illness, 2) learning about ART and overcoming barriers, 3) parenting and maintaining a healthy home, 4) how to improve coping (i.e., religion, stigma and care for family), 5) basics of good nutrition (i.e., cooking tips), and 6) life skills class (i.e., computers, embroidery, etc). In addition, the WLHA received monthly supplies of protein supplementation.^36,43^ On the other hand, the Usual Care (UC) program included 6 group sessions focused on the importance of adherence, parenting, keeping healthy, basic nutrition, and physical and psychological assessment; however, there was no ASHA support.^36^ The findings suggest that the AL intervention improved depressive symptoms,^43^ stigma and avoidant coping,^44^ adherence,^45^ and physiological characteristics (ie, body mass index, muscle mass, fat mass, ART adherence, and CD4 counts) among rural WLHA^46^ as compared to the UC program.

In a qualitative study conducted among WLHA who participated in the AL study in Nellore, Andhra Pradesh, depressed mood and anxiety were experienced prior to their participation.^47^ Further, participants reported how the program was helpful in managing mental health symptoms and the role of ASHA and staff in providing support with mental health symptoms.^47^ Data also revealed the importance of tangible support, need for social support, and their experience of having difficulty accessing ART.^36^

### Statement of Problem

While literature has explored interrelationships between mental and physical health symptoms, nutrition, stigma, and discrimination, there is a paucity of data to understand if extant findings can be applicable to rural WLHA in Prakasam, Andhra Pradesh, India. In particular, this district is a drought prone and has high levels of poverty.^31–33^ Accordingly, rural WLHA may experience different challenges; therefore, we aim to understand how to tailor the intervention for this community.

### Purpose

The purpose of this qualitative study was to conduct focus groups with a small sample of WLHA from Prakasam to tailor a future intervention for this vulnerable population.

## Methods

The following paragraphs have been guided by the consolidated criteria for reporting qualitative research, which include the following areas: (1) study design, (2) research team and reflexivity, and (3) data analysis and reporting.^48^

### Design

As part of a 2-phase study, qualitative methods which utilized focus groups were guided by a semi-structured interview guide (SSIG) to understand perspectives among rural WLHA (N = 24) residing in Prakasam, Andhra Pradesh, India.

### Site

Participants from 4 sites located within Prakasam were recruited into the study. Prakasam is a rural district and impoverished area in the state of Andhra Pradesh, India, characterized by seasonal drought.^31–33^

### Sample

The eligibility criteria were as follows: (1) 18 to 55 years of age and (2) currently receiving ART. Prior to implementation, both domestic and international ethics committees approved this study, which include UCLA Institutional Review Board for Human Subjects Protection and All India Institute of Medical Sciences.

### Procedures

After required regulatory approvals were obtained, research staff informed women about the study by flyers distributed in the primary health center (PHC) and by a brief presentation. The brief presentations described the study and questions were entertained and answered about participation in the focus group sessions (FGSs). Persons who expressed interest in the study and provided brief oral consent were assessed for eligibility in a private area of the PHC. Among women who were interested, a brief screener was administered, which asked questions regarding age and current use of antiretroviral therapy.

Once participants were screened as eligible, a waiver of informed consent was administered (ie, study information sheet) and included information related to the study purpose, procedures, and potential risks as well as benefits. Appointments were set for the discussions to be in Telugu or English and occurred in a private area of the PHC. Each FGS enrolled 6 to 8 participants and was led by the project manager (PM) who was fluent in Telugu and English. The PM has over a decade of experience with international projects in this role and she has coauthored a number of studies with the research team.^36,42,47^

The SSIG, used to guide the discussion, included open-ended questions related to nutritional issues and ongoing barriers to ART. In particular, the SSIG focused on topics such as health services sought, access to care, and HIV/AIDS program resources needed. Relatedly, the SSIG also assessed preferences on how care should be delivered. In total, 3 tape-recorded FGSs were conducted. For their participation, each participant received ₹ 525 (US$10) after the FGS.

### Data Analysis

**Figure 1.**
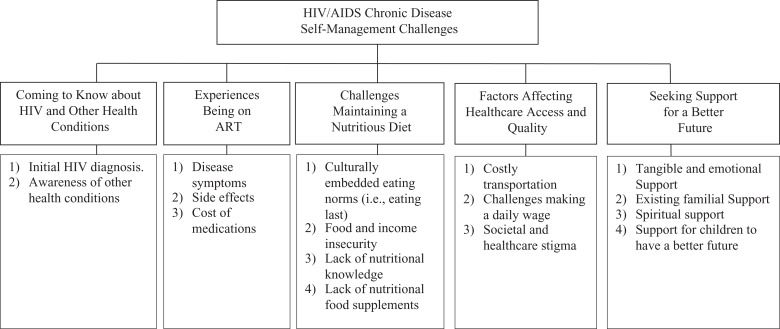
Chronic disease self-management challenges among rural women living with HIV/AIDS in Prakasam, Andhra Pradesh, India (N = 24).

Sample characteristics were described by frequencies, percent, and means. Four research team members were involved in the data analytic process. Naturalistic inquiry was ensured by applying the principles of (1) confirmability, (2) dependability, (3) transferability, and (4) credibility.^49^ During the data analytic steps, the software utilized included Microsoft Word, PowerPoint, and Dedoose.^50^ Throughout the data analytic steps, the research team used PowerPoint to develop and draw themes, and the process that emerged from the data is depicted in Figure 1. An audit trail detailing specific steps was established for confirmability to be ensured.^49^ Audio recordings were saved and the project director transcribed the audio files from Telugu to English.

The data analytic process included the following steps: (1) precoding, (2) initial line-by-line coding, (3) examining cultural domains, and (4) using eclectic coding methods.^51^ The research team compared codes based on similarities, differences, and categories emerged.^51^ Theming the data led to higher level constructs and themes^52^ which were clustered together. Supporting themes were clustered based on repetitions (eg, reoccur with regularity), similarities, and differences.^52^ These findings were shared with the community and researchers to obtain feedback.

## Results

Table 1 reports sociodemographic characteristics of the WLHA sample (N = 24, age range = 20-42; standard deviation = 4.55). An equal number of women had 1 or 2 children (48.5%); fewer women reported having 3 children (8.3%). The majority of the participants was married (66.7%) or widowed (29.2%). Figure 1 depicts 5 major theoretical constructs, which emerged from the data such as (1) coming to know about HIV and other health conditions, (2) experiences being on ART, (3) challenges maintaining a nutritious diet, (4) factors affecting health care access and quality, and (5) seeking support for a better future.

**Table 1.** Sociodemographics of Rural Women Living with HIV/AIDS in Prakasam, Andhra Pradesh, India.^a^

	Mean	SD (range)
Age	30.25	4.55 (20-42)
	n	%
Children		
1 child	11	45.8%
2 children	11	45.8%
3 children	2	8.3%
Marital status		
Married	16	66.7%
Widowed	7	29.2%
Separated	1	4.2%

Abbreviation: SD, standard deviation.

^a^N = 24.

### Theme 1: Coming to Know about HIV and Other Health Conditions

Rural WLHA described when they became aware of their HIV diagnosis and other health conditions. The majority of women learned of their HIV diagnosis while they were pregnant or had a newborn diagnosed with HIV. Other rural WLHA learned about their HIV diagnosis when their spouse became ill. One woman expressed:

The first time when I knew I was HIV positive, I was pregnant at that time and also having high fever. But, fortunately, my child is not having HIV. (Participant P)

While another woman described the following:

Three years back, I knew about my HIV status when my husband had [a] skin infection. He got treatment from a private doctor and was cured. After that incident, we both were on ART medications. (Participant N)

Rural WLHA expressed that they had other health conditions, which included tuberculosis (TB) and/or cervical cancer. One woman shared:

My husband was very sick, and at that time I…also tested [positive for] HIV. I had TB and started ART. I became very weak and thin. (Participant O)

Another woman described the symptoms she was experiencing and her diagnosis of cervical cancer. She said:

I am having severe white discharge and doctors told me that I am suffering with cervical cancer. (Participant G)

### Theme 2: Experiences Being on ART

Several women shared their experiences being on ART, which included disease symptoms, side effects, and the cost of the medications. One woman shared:

I am on ART and I missed my pills often…Me and my husband [are] having TB. When the weather is very cold, we get very sick. I also have fevers, [and] severe cough. (Participant U)

Another woman discussed her appetite and feelings of being emotionally affected. She said:

I don’t feel hungry enough and lose interest to access health care or taking my ART pills. This also…impacts on my parenting. In a week…[I feel mentally ill every…] 2 to 3 days. (Participant X)

For several women, physical symptoms resulted in missing ARV doses.

When [my] husband died, my child tested positive for HIV. I also feel very sick often…with diarrhea [and] fevers. Most of the time, I…[am] unable to take ART due to mental and physical sickness. (Participant V)

Side effects of the medication were also commonly reported.

My ART drug…[is] always making [me] feel sleepy, drowsier, unable to concentrate, [and] mental disturbances. (Participant E)

Another WLHA expressed the following:

Because of my side effects, I also suffer with [infections] and mental restlessness and fight with everybody I live with. (Participant A)

Another woman shared that after an assessment at the District Hospital (DH) for severe stomach pain, the physician requested that she purchase medications from outside the DH; however, she was unable to afford that medication. She described:

I am a widow and unable to buy second-line drugs so I applied for receiving free second-line drugs here in the DH. (Participant H)

### Theme 3: Challenges Maintaining a Nutritious Diet

Women described challenges with maintaining a nutritious diet. These included culturally embedded eating norms (ie, eating last), food and income insecurity, lack of nutritional knowledge, and lack of food supplements (ie, nutritional supplements). Across all of the focus groups, women described having to eat last after the husband and other family members, when the food quantity was minimal. One woman shared, “I receive…less food because I eat at the end.” (Participant X)

All rural WLHA shared that they did not have enough food once or twice a week, and for others, once or twice a month. One woman described the interrelationships between eating, taking medication, and adherence with her ART. She said:

If we do not have food, we miss our pills because if I take ART without food, I feel giddy and sick. So, whenever I am not eating enough food, I miss my pills. We need guidance, support, food, and information on food. We need foods like milk, eggs, and pulses. (Participant P)

While other participants reported the relationship between living in a drought area, food, and financial insecurity:

We carry drinking water from far place for daily use. When we are sick, we do not work and do not have enough money to buy food. (Participant T)

While another WLHA said:

We need food supplement. Our area is drought prone so we have less water access and also less work. (Participant V)

Women also described a lack of nutritional knowledge in general and about supplements. One woman shared:

We need help to be healthy…We need more health information and support for being healthy. (Participant E)

### Theme 4: Factors Affecting Health care Access and Quality

Rural WLHA described a number of factors affecting health care access such as costly transportation, challenges making a daily wage, and societal and health care stigma related to HIV-positive serostatus. Rural WLHA described their need for more advanced specialists such as dermatologists (eg, skin experts), psychiatrists, and gynecologists. They also described challenges accessing basic and specialty care. For some rural WLHA, seeking treatment for minor illnesses from local village “*quacks*” was more possible because the cost of travel to reach DH from the village was very expensive. For illnesses that were more serious, they went to the DH. However, the cost of health care was a determining factor, which affected access to medication. In particular, one woman shared:

To go to private hospital is very expensive which we could not afford. The DH is prescribing some medications which we need to buy from outside. Only very few medicines are supplied here in DH. I request the government to give all medications from the DH’s pharmacy…(Participant N)

Other women shared that they often do not go to the hospital but would appreciate treatment within the village or with a shorter travel distance. One woman shared:

Because I was sick, I visited the…government hospital. I would appreciate treatment within the village or with lesser distance. (Participant X)

Some women shared that they needed transportation to access the hospital and also needed child care. In particular, one woman added:

We need food, transportation, and better treatment and services for my diseases…(Participant C)

Women also shared that being ill contributed to not making a daily wage; thus, they were not able to pay for health care. In particular, one woman expressed:

When I am sick, I lose my daily wage and I will not be able to pay for my travel to visit hospital and also medications or even buy food for the family and me. (Participant K)

Across the focus groups, rural WLHA shared their experiences with HIV-related stigma being present not only in their community but also among health care providers. This impacted the quality of health care received. The psychological outcomes of feeling stigmatized were described by rural WLHA as feeling “separate,” “feeling bad,” and being “mentally sick.” In addition, some rural WLHA shared that they felt that people did not even touch them. One woman shared her community experience:

Even collecting ART medications and using them is also a problem, fearing people will find out about our HIV status and stigmatize us. This is stopping us to [be] 100% adherent. We are troubled due to societal stigma. Stigma and sickness are the major problems for me. (Participant M)

However, stigma was not only limited to the community setting, some women described observing practitioners who responded negatively to nondisclosure.

Once I came here to this hospital for severe white discharge problem and I saw the physician shouting on that women for not revealing her health status, so I was afraid to talk about my status. (Participant X)

Another woman further shared that treatment was limited for those who had an HIV diagnosis.

When we are having a major health problem which requires surgery. The government hospital does not do any surgeries especially they refuse persons living with HIV. (Participant N)

### Theme 5: Seeking Support for a Better Future

Rural WLHA discussed that they were interested in support, which included tangible and emotional support, existing familial support, spiritual support, and support for their children to have a better future. Rural WLHA reported a desire for tangible and emotional support from more women like themselves, group sessions, and individuals to help “boost up their confidence.” Women also articulated their interest in garnering skills to assist them in “standing on their feet.” One woman shared:

We need support for food and transportation and good health care services and my child needs good health. (Participant S)

Women appreciated the opportunity to share how they felt in the FGSs and found the time in the session beneficial. One woman expressed:

Group meeting like the one we are having now. We never experienced a session like this. No one ask us how we are feeling like the way you are asking. We need sessions like this and facilities for better health. (Participant X)

Other rural WLHA revealed that assurance and counseling were important ingredients in their interactions. One woman shared:

…There are many persons like us [who are] less confident and live in fear. We need someone to assure us that everything will be alright. We need home counselling. Our confidence levels have to be boosted up. (Participant N)

Women shared that spousal support was important and often it was a member of the family that helped remind them of taking pills. One woman reported:

Most of the time, I miss my pills. My husband reminds me. Due [to] my mental health, I miss [taking my pills]. (Participant K)

Challenges maintaining good relationships were shared among participants and others described how some close relationships ended. One participant reported that being HIV positive affected her relationship with her husband. She said:

My husband left me when he knew that I was HIV positive. I have no support and I live with my kids. (Participant P)

Across all 3 FGSs, rural WLHA wanted their children to have good health and get well soon. Others shared that their children were not well. One woman described how their mental health was affected due to concern for the welfare of their children and future. One woman articulated how her children’s health affected her, “I always fear what happens to my children; this affects my mental health a lot.” While another shared that seeing young children in the ART was depressing. Across the focus groups, women mentioned a desire for a permanent cure for HIV.

## Discussion

The purpose of this qualitative study was to explore the perspectives voiced by rural WLHA in Prakasam, Andhra Pradesh, India. Our findings suggest that rural WLHA are experiencing a myriad of CDSM challenges, which include adhering to ART, managing a nutritious diet, and accessing quality health care. Rural WLHA also shared their experience coming to know their HIV diagnosis.

Rural WLHA described their challenges being on ART, which included managing symptoms. For several of the women, they described an inability to take their ARV medications due to mental and physical illness. Rural WLHA described physical symptoms that they were experiencing, which included body pains and weakness, heavy vaginal bleeding, vomiting and abdominal pain, and white discharge. Some rural WLHA may attribute some of these symptoms to being on ART, while others described their symptoms. Another important finding is the mental health symptoms, which included feeling mentally ill which sometimes precluded taking their ARV medications. Other researchers have found high levels of depression among PLWH or patients taking ARV medications.^38,53^ In a Delhi-based study, depressive symptomology was present in over half the sample (58.8%).^53^ These findings point to the need to conduct screening for depression and provide depression-specific care among rural WLHA.

Across the focus groups, our findings detailed challenges WLHA experienced maintaining a nutritious diet. These challenges were found in culturally embedded eating norms, which included eating less as compared with others in their family. In Indian culture, the role of women in preparing food is an integral part of the household; in fact, some authors charge that there is no substitution for a woman in the kitchen as she provides a “special sweetness” to foods.^54^ The woman of the house first serves the food to the guests, oldest men, all male diners, then the children, and women.^54^ Thus, due to this culturally embedded eating and ordering,^54^ it is likely that she eats last and less food than anyone else. Future research should focus on the importance of the WLHA to receive adequate nutrition, as it will influence her ability to manage HIV and other comorbid conditions adequately.

Rural WLHA shared not having enough food was a deterrent to medication adherence because taking ARV medication without food led them to feel mentally ill. Women shared the interaction between the environment and food availability; in particular, rural WLHA shared that due to the drought, there was less water access and work. In the coastal region of Prakasam, drought is common; in fact, the Prakasam economy is primarily dependent on agriculture.^31^ Given these findings, one method may be to encourage home gardens^55^ whereby women would be able to plant their own edible food.

Due to feeling ill, rural WLHA reported that they missed work at times; likewise, they did not seek treatment or receive enough food during those times. As stated earlier, rural WLHA often skipped ARV medication when food was not readily available. This cyclical pattern of ability to work and food security influences ARV medication adherence. Thus, the request by the WLHA for support in the form of food supplementation, transportation to health care services, and better access to quality health care services is critical to support the women in adherence to ART and optimum health.

In a Chennai-based qualitative study, similar themes, which emerged, included barriers to ART that were illness, psychological, financial, and transportation related.^30^ The geographical drought-stricken area of Andhra Pradesh further leads to challenges with obtaining nutritious foods. Rural WLHA also shared that they needed specialty health care providers such as dermatologists and gynecologists. Too often, village “quacks” were more accessible to the rural residents due to not having adequate access to providers.

Rural WLHA also described that they perceived others (ie, such as health care providers) stigmatized them in their communities. For some, HIV-related stigma challenged their ability to access HIV treatment due to maltreatment in their homes, village, and community setting.^36^ Critical strategies that can assist women in learning to cope with stigma include finding a supportive network. Providing solace and comfort with the women would then enable them to focus on their children, as they wanted the best future for them.

### Study Limitations

This study has several limitations that are important to discuss. First, this was a convenience sample of women aged 20 to 42 and is limited to the Prakasam District in Andhra Pradesh, India. Likewise, the sample size may be too small to generalize findings outside of this rural area and among women beyond this age span or who do not have HIV/AIDS. These findings represent the perspective of the population and it is plausible that participants may not be knowledgeable of all the resources in the community. Further, social desirability bias in which women may want to please the research team with aspects they feel they want to hear may be operating.

Relatedly, the first author of the article, research team, and the principal investigator (PI) of the study have all engaged with the English language version of the transcript and not the Telugu version which may not capture culturally specific or sensitive findings. However, to address this concern, our Indian-based research team reviewed the manuscript and findings. Further, the first author has coded the data with the research team from an outsider’s perspective. Correspondingly, the first author has coauthored previous studies among rural WLHA.^43–45^

### Nurse-Led Program Development Implications

Rural WLHA experience CDSM challenges, which include difficulty managing symptoms, a nutritious diet, and psychological well-being. Not only are rural WLHA managing their own HIV-positive status, but they are also managing their families’ health, physical, and mental well-being simultaneously. As an overlay, the cultural context of being a wife, mother, and caregiver while trying to self-manage their own chronic disease process challenges their quality of life, endurance, and ability to self-care.

Further, challenges in a drought-prone, rural environment, access to health care providers and cost of transportation make it very difficult for rural WLHA to obtain the appropriate tangible and emotional support to self-manage their chronic condition in a rural locale. Further, stigmatization, which is felt and perceived by their community and health care providers, is another critical component, which can be internalized and affect their willingness to seek out health care. Given these findings, we believe that formative qualitative work related to the meaning and concept of “*self-care*” needs to be deconstructed among rural WLHA as it may provide insight into perspectives, needs, and opportunities to gain a greater understanding of familial, self, health, and societal priorities. It is important to consider that in this culture, “*self-care*” may be inclusive of the family unit and not necessarily focus solely on the “*self*.”

Based on our emergent findings, previous work and the multidisciplinary team that developed this current study necessitates a nurse-led and ASHA-partnered intervention, which addresses culturally sensitive, self-management skills, which includes understanding and having access to an appropriate diet, ability to maintain ARV medication adherence, and providing positive coping mechanisms. Our previous work has focused on integrating ASHA into the delivery of care for HIV-positive rural women.^43–45^ The ASHA role was filled by women who had at least high school education, had an interest in caring for WLHA, and lived in the same village.^45^ Our data revealed that there was a significant increase in ART adherence in the AL intervention group as compared with the control group.^45^

Based on our most recent qualitative findings in Prakasam and in consideration of our previous research findings, it is critical to incorporate both nurses and community health workers (CHWs) or ASHA to deliver the intervention taking into account the family unit, along with providing food supplementation, the provision of emotional support, and assistance with transportation and health care access. Previous research has utilized a combination of nurses and CHWs in the provision of health services^56,57^; this cost-effective model can be applied and tailored in a culturally sensitive manner to rural WLHA. The purpose of using this model is to provide self-management support utilizing a health care provider and CHW equivalent, namely ASHA. The ASHA will be able to serve as a liaison between health care providers and rural WLHA. Relatedly, the nurse will be able to provide greater care for rural WLHA, serve as a liaison between DH, and utilize the support of ASHA.

While CDSM in rural locales is challenging given the number of barriers experienced by WLHA, these findings encourage the development of both individual- and structural-level support systems that might be helpful in assisting rural WLHA to self-manage HIV as a chronic illness for themselves and their families.
